# Mobilization of CD34+CXCR4+ Stem/Progenitor Cells and the Parameters of Left Ventricular Function and Remodeling in 1-Year Follow-up of Patients with Acute Myocardial Infarction

**DOI:** 10.1155/2012/564027

**Published:** 2012-03-28

**Authors:** Rafał Wyderka, Wojciech Wojakowski, Tomasz Jadczyk, Katarzyna Maślankiewicz, Zofia Parma, Tomasz Pawłowski, Piotr Musiałek, Marcin Majka, Marek Król, Wacław Kuczmik, Sebastian Dworowy, Barbara Korzeniowska, Mariusz Z. Ratajczak, Michał Tendera

**Affiliations:** ^1^Third Division of Cardiology, Medical University of Silesia, 45-47 Ziołowa Street, 40-635 Katowice, Poland; ^2^Institute of Cardiology Jagiellonian University, John Paul II Hospital, Pradnicka 80, 31-202 Krakow, Poland; ^3^Department of Transplantation, Jagiellonian University, Wielicka 265, 30-663 Krakow, Poland; ^4^American Heart of Poland, Sanatoryjna 1, 43-450 Ustroń, Poland; ^5^Division of Vascular Surgery, Medical University of Silesia, 45-47 Ziołowa Street, 40-635 Katowice, Poland; ^6^Stem Cell Institute, University of Louisville, 2301 South 3rd Street, Louisville, KY 40208, USA

## Abstract

Mobilization of stem cells in acute MI might signify the reparatory response. *Aim of the Study*. Prospective evaluation of correlation between CD34+CXCR4+ cell mobilization and improvement of LVEF and remodeling in patients with acute MI in 1-year followup. *Methods*. 50 patients with MI, 28 with stable angina (SAP), and 20 individuals with no CAD (CTRL). CD34+CXCR4+ cells, SDF-1, G-CSF, troponin I (TnI) and NT-proBNP were measured on admission and 1 year after MI. Echocardiography and ergospirometry were carried out after 1 year. *Results*. Number of CD34+CXCR4+ cells in acute MI was significantly higher in comparison with SAP and CTRL, but lower in patients with decreased LVEF ≤40%. In patients who had significant LVEF increase ≥5% in 1 year FU the number of cells in acute MI was significantly higher versus patients with no LVEF improvement. Number of cells was positively correlated (*r* = 0,41, *P* = 0,031) with absolute LVEF change and inversely with absolute change of ESD and EDD in 1-year FU. Mobilization of CD34+CXCR4+ cells in acute MI was negatively correlated with maximum TnI and NT-proBNP levels. *Conclusion*. Mobilization of CD34+CXCR4+ cells in acute MI shows significant positive correlation with improvement of LVEF after 1 year.

## 1. Background

Small numbers of bone-marrow (BM-) derived stem and progenitor cells (SPC) are present in peripheral blood in humans. In acute coronary syndromes (ACS) and stroke the number of circulating cells significantly increases. Such mobilization of SPC is an inflammatory reaction, but the presence of primitive SPC can also reflect the reparatory mechanism. Mobilization of endothelial progenitor cells (EPCs) reflects the turnover of vascular endothelial cells, because these cells contribute to endothelial renewal [1–3]. Myocardial infarction (MI) triggers the mobilization of not only EPCs, but also other populations such as hematopoietic stem cells (HSCs), mesenchymal stromal sells (MSCs), very small embryonic like cells (VSELs) and other less well-defined types [4, 5]. One of the populations that undergoes rapid mobilization in acute MI are cells expressing chemokine receptor CXCR4. These cells are enriched for early markers of myocardial and endothelial differentiation and in part also markers for primitive embryonic-like stem cells (Oct-4, SSEA-4, Nanog) [5]. Our previous studies demonstrated that in acute MI within several hours after the onset of the chest pain there is a robust increase of CD34+CXCR4+ and CD34+CD117+ cells. The mobilization coexists with significant upregulation of cardiac (GATA-4, Nkx2.5/Csx, MEF2C) and endothelial lineage markers (VE-cadherin, von Willebrand factor), which suggests that these cells might contribute to tissue repair following ischemic injury [4]. Mobilization of BM-derived SPC is regulated by chemoattractants released by ischemic myocardium, complement cascade, and bioactive phospholipids [6].

In particular stromal-derived factor-1 (SDF-1)–CXCR4—axis might contribute to homing of the SPC to the infarct border area in the heart where it is expressed following MI. This signaling axis is also the key factor regulating the mobilization of BM cells and renewal of hematopoiesis as well as in inflammation [7]. Mobilization of BM by G-CSF is mediated by disruption of SDF-1-CXCR4 binding [8]. Increased production of SDF-1 via activation of hypoxia-inducible factor 1-*α* within the ischemic myocardium facilitates the homing and engraftment of circulating BM cells which subsequently participate in the reparatory processes [9].

Mobilization of SPC was investigated as a potential prognostic marker in patients with stable coronary artery disease (CAD) and the number of circulating EPCs correlated with CAD risk factors, endothelium-dependent vasomotion, and risk of ischemic events [10–12].

Prognostic value of measurement of SPC mobilization in ACS is less well known. Acute MI triggers substantial inflammatory response which might affect the mobilization and trafficking of stem cells. In addition, intensive treatment with drugs known to affect the SPC release from the BM such as statins and ACE-I might modulate to mobilization and migration intensity. Other important factors are patients age and comorbidities in particular diabetes [13]. There is a paucity of data on the association between mobilization of SPC which might contribute to myocardial tissue repair and the improvement of the left ventricle (LV) contractility and remodeling; however, pilot studies showed that in patients with reduced LVEF in acute MI the mobilization of cells is less efficient [14].

Improvement of LVEF following the primary percutaneous coronary intervention (pPCI) is a positive prognostic factor for long-term survival in acute MI. Spontaneous mobilization of SPC in acute MI is a form of reparatory mechanism; therefore we conducted a prospective study to evaluate the relationship of CD34+CXCR4+ cell mobilization and long-term recovery of LV contractility, remodeling, and clinical status (ergospirometry, NYHA, CCS class) in patients with acute MI in 1-year follow-up.

## 2. Patients and Methods

Study population consisted of 98 patients: 50 patients with acute myocardial infarction (MI), 28 patients with stable angina pectoris (SAP), and 20 individuals with no history of ischemic heart disease (control group, CTRL). Subjects with myocardial infarction were diagnosed according to the current ST-elevation myocardial MI (STEMI) definition.

Inclusion criteria for patients with myocardial infarction were

1. time interval between the onset of chest pain and hospital admission <12 hours,
2. age < 75 years,
3. patients qualified to pPCI.

Abciximab was administered in 64% of patients during PCI procedure. All patients received unfractionated heparin (70 U/kg) to achieve ACT values >250. In all patients TIMI3 flow in the infarct-related artery was achieved. Statins (67% simvastatin and 33% atorvastatin) were administered starting from the first day of hospitalization.

Exclusion criteria were

1. history of MI in the past,
2. cardiogenic shock (IV class according to Killip-Kimball scale),
3. neoplastic disease,
4. kidney and/or liver failure,
5. coagulopathies and/or hematopoietic system diseases,
6. autoimmunological disorder and/or systemic inflammatory process,
7. history of surgical procedure or coronary arteries percutaneous intervention (revascularization) within last 6 months.

Patients were diagnosed to have stable angina pectoris according to the following: (a) typical clinical presentation/symptoms (chest or arm discomfort/angina reproducibly associated with physical exercise), (b) noninvasive test (positive exercise test/treadmill stress test) and qualified to planned coronarography. Presence of ≥1 significant stenotic lesion (≥70%) in coronary arteries was reported. Stable angina pectoris (SAP) and acute myocardial infarction (AMI) groups were matched to avoid major differences in the context of risk factors and pharmacological treatment which may affect the number of cells circulating cells.

Control group (CTRL) individuals were diagnosed due to valvular heart disease or rhythm disturbances.

The study protocol was approved by the Ethics Committee of the Medical University of Silesia and all patients signed informed consent. The study conformed to the Declaration of Helsinki and was funded by the European Union structural funds—Innovative Economy Operational Programme, Grant POIG.01.01.02-00-109/09 “Innovative methods of stem cells applications in medicine” and Polish Ministry of Science and Higher Education Grants 0651/P01/2007/32, 2422/P01/2007/32 and statutory funds of Medical University of Silesia. 

### 2.1. Laboratory Measurements

Peripheral blood (PB) samples were collected within 12 hours of the first symptoms and 1 year after in patients with myocardial infarction, in SAP and control group during routine clinical follow-up visit. 4–6 mL of PB was obtained from each patient and stored in both vacuum heparin tubes (2-3 mL; measurement of progenitor cell number) and vacuum EDTA tubes (2-3 mL; measurement of hematopoietic cytokines concentration).

The following parameters were measured:

1. number of CD34+/CXCR4+ progenitor cells,
2. concentration of chemoattractant factors (SDF-1, G-CSF),
3. troponin I (TnI) concentration and creatine kinase MB isoenzyme (CK-MB) activity,
4. NT-proBNP and high sensitive C-reactive protein (hsCRP) concentration.

#### 2.1.1. Measurement of CD34+CXCR4+ Cells

Blood samples were transported in 4°C to FACS facility processed within 4–6 hours after drawing. CD34+CXCR4+ cells number was analyzed with FACS based on specific membrane antigens expression in accordance to the ISHAGE criteria (International Society of Hematotherapy and Graft Engineering) [15]. For isolation of mononuclear cells (MNCs) samples were centrifuged through a Ficoll density gradient and subsequently suspended in phosphate-buffered saline (PBS) (1 × 10^5^/100 uL). Afterwards, MNCs were stained with fluorochrome-conjugated mouse monoclonal antibodies (Abs) for the CD34 (phycoerythrin- (PE) conjugated Abs) and CXCR4 (allophycocyanin- [APC-]conjugated Abs) and isotope control (BD, Pharmingen, San Diego, CA, USA). Staining was performed at 4°C for 30 minutes without light exposure. Cells were subsequently washed twice in PBS, resuspended in 200 *μ*L of PBS, and analyzed using a flow cytometer (FACSCalibur, Becton Dickinson, San Jose, USA). At least 106 events were acquired from each sample. The percentage content of CD34+/CXCR4+ cells was calculated with appropriate isotope control cut-offs. The absolute number of (cells/*μ*L) was calculated according to the previously published method: CD34+/CXCR4+ percentage × leucocytes number/100 [14].

#### 2.1.2. Chemoattractant and Inflammatory Markers

Collected PB samples were centrifugated (1000 ×g) at 4°C for 15 minutes. Obtained plasma was stored at −30°C. The centrifugation was performed within 30 minutes from blood sampling. Additionally, for SDF-1 level measurement samples were centrifuged (10 000 ×g) for 10 minutes in order to eliminate platelets. Plasma levels of SDF-1, G-CSF, NT-proBNP, and C-reactive protein were quantified using high sensitive kits (G-CSF (Bender Medsystems); SDF-1 (Quantikine, R&D systems); NT-proBNP (Quantikine, R&D systems), hsCRP (Behring Nephelometer II Dade Behring)).

### 2.2. Echocardiography

Echocardiography was performed after admission to hospital (<12 hours of chest pain symptoms) and 12 months post discharge during the follow-up visit by experienced echocardiolographer. Transthoracic echocardiography (M-mode and typical 2D projections) was carried out in accordance to the American Society of Echocardiography guidelines.

Evaluated echocardiography parameters were: left ventricle end-diastolic (EDD) and end-systolic (ESD) diameter and left ventricle ejection fraction (EF%) according to Simpson method.

### 2.3. Ergospirometric Test

The test was performed 12 months after myocardial infarction on the treadmill according to modified Bruce protocol. The following parameters were analyzed: resting heart rate (HRrest), peak heart rate (HRpeak), maximum exercise time (*T*max), energy expenditure in METs, maximal exertional oxygen uptake (VO_2_ peak) presented as mL/kg/min and percentage of calculated norm (VO_2_ peak %N), resting and peak ventilatory equivalent for oxygen (VE/VO_2_ rest, VE/VO_2_ peak), peak/resting ventilatory equivalent for oxygen ratio (VE/VO_2_ peak/rest), resting and peak ventilatory equivalent for carbon dioxide (VE/VCO_2_ rest, VE/VCO_2_ peak), peak/resting ventilatory equivalent for carbon dioxide ratio (VE/VCO_2_ peak/rest), ventilation relative to carbon dioxide production (VE/VCO_2_ slope), oxygen pulse and heart rate reserve (HRR)—according to the American College of Sports Medicine guidelines and methods. Test was continued until limiting symptoms (fatigue, chest pain, dyspnea) or lack of VO2 increase occurred. Test was carried out on Oxycon Delta (Jaeger) system.

### 2.4. Clinical Status

Heart failure symptoms were evaluated according to New York Heart Association (NYHA) classification and angina severity according to Canadian Cardiovascular Society (CCS) class.

### 2.5. Statistical Analysis

Number of SPC and levels of chemoattractants were expressed as median and interquartile range (IQR). *U* Mann-Whitney and Wilcoxon tests were used for comparison of time points and groups and Spearman rank test for assessment of correlation. Logistic regression was used to identify the factors associated with significant (2-fold) mobilization of cells. Value of **P** < 0.05 was considered significant. Statistica 6.0 PL for Windows package was used.

## 3. Results

Study groups (AMI and SAP patients) were comparable with respect to risk factors profile, demographic data, and laboratory results excluding leucocyte number which was statistically significantly higher in MI group. In comparison to SA group patients with MI less frequently were on chronic treatment with ASA (*n* = 31 (62%) versus *n* = 28 (100%), *P* < 0.05). In MI group anterior MI was diagnosed in 30 (60%) and multivessel coronary disease in 26 patients (52%). In comparison of MI and SAP group with CTRL group the following parameters were statistically significantly higher in the study groups: mean age, percentage of patients with hypertension, hypercholesterolemia, type 2 diabetes mellitus, and family history of ischemic heart disease and smoking. Clinical and demographic characteristics of the study population is shown are Table 1. 50 patients were followedup 1 year after MI. The medical treatment at the time of followup consisted of ASA (all patients), statin (*n* = 49), ACEI (*n* = 48), and beta-blockers (47 patients).

### 3.1. Mobilization of Stem/Progenitor Cells

**Figure 1:**
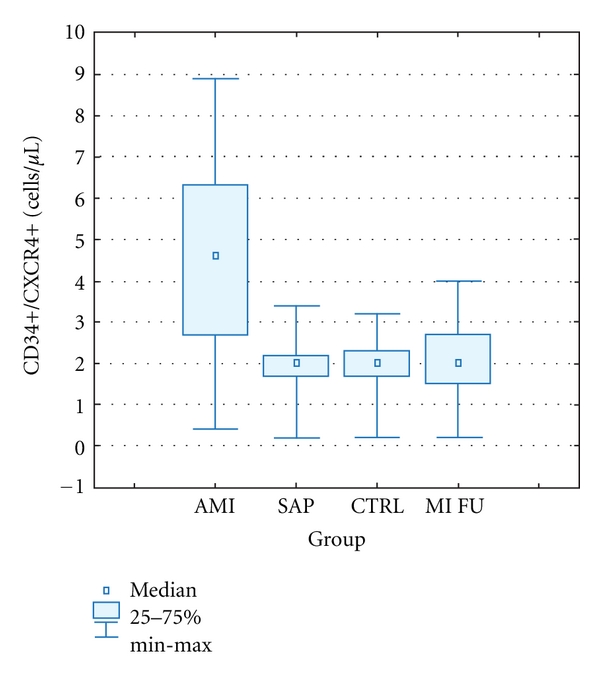
The number of circulating progenitor CD34+/CXCR4+ cells in peripheral blood. AMI: acute myocardial infarction, SAP: stable angina pectoris, CTRL: control group, MI FU: 1-year followup. Data is presented as cell number in 1 *μ*L of peripheral blood (median ± IQR). CD34+CXCR4+: CTRL 2,0 (0,2–3,4); SAP 2,1 (0,2–3,2); AMI 4,6 (0,4–8,9); MI FU 2,2 (0,2–4,0).

The absolute number of CD34+CXCR4+ cells in patients with acute MI was statistically significantly higher in comparison with SAP and CTRL groups. In 1-year followup the number of circulating CD34+CXCR4+ cells was similar in all three groups. There were no statistically significant differences in cell number between the control group and stable angina pectoris group (Figure 1).

No differences in stem cell mobilization were noted in subgroups of patients (males versus females (2,3 (0,3–8,95) versus 2,1 (0,1–8,5); *P* = 0.99], presence of type 2 diabetes [2,4 (0,1–7,6) versus 2,1 (0,1–8,9); *P* = 0.31). We found also no significant differences in patients who were on chronic treatment with statins [2,7 (0,4–7,9) versus 2,1 (0,4–7,9); *P* = 0,84], ACE-I [2,1 (0,4–7,8) versus 2,4 (0,1–8,9); *P* = 0,58].

### 3.2. Levels of Chemoattractants

**Figure 2:**
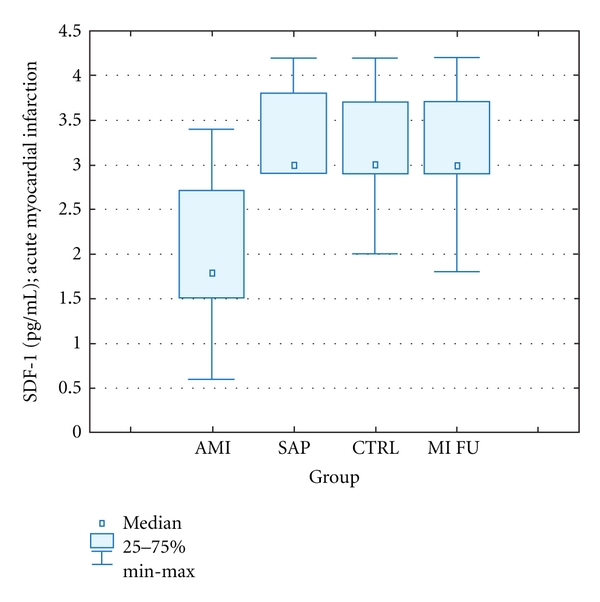
PlasmaSDF-1 levels. AMI: acute myocardial infarction, SAP: stable angina pectoris, CTRL; control group, MI FUL: 1-year followup. Data is presented as cell number in 1 *μ*L of peripheral blood (median ± IQR).

In patients with acute MI peripheral blood SDF-1 concentration was significantly lower than in SAP patients and healthy individuals. In 1-year follow-up, there was no difference in plasma SDF-1 level among three groups. No significant differences in SDF-1 concentration between CTRL and SAP group were observed (Table 2, Figure 2).

**Figure 3:**
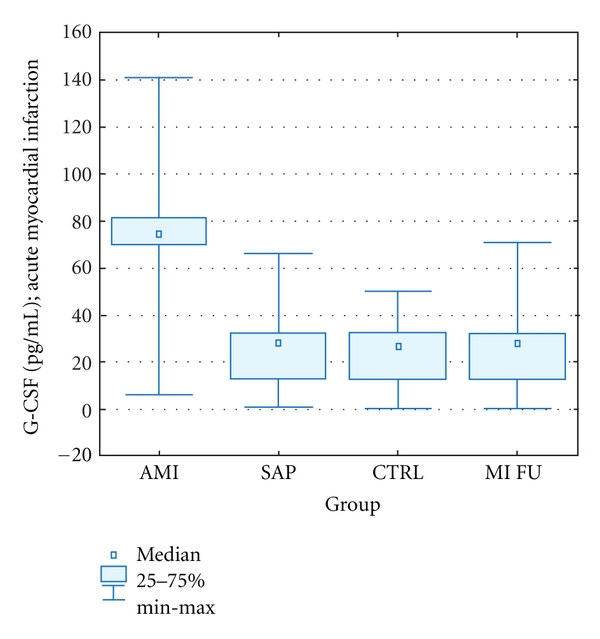
Plasma G-CSF levels. AMI: acute myocardial infarction, SAP: stable angina pectoris, CTRL: control group, MI FU: 1-year followup. Data is presented as cell number in 1 *μ*L of peripheral blood (median ± IQR).

In patients with MI G-CSF concentration was significantly higher comparing to SAP and control group. No significant differences in G-CSF concentration between CTRL and SAP group were observed. In 1-year followup, plasma G-CSF level was similar in all three groups (Table 2, Figure 3).

In AMI study group there was significant positive correlation between SDF-1 level and mobilized CD34+/CXCR4+ cells number (*r* = 0, 41, *P* = 0,023). After 1 year, there was no significant correlation between levels of SDF-1 and G-CSF and number of circulating cells. We found no differences in SDF-1 and G-CSF levels in subgroups of patients (males versus females, presence of type 2 diabetes, and chronic treatment with statins). In patients with MI older than 50 years the number of mobilized CD34+CXCR4+ cells and plasma SDF-1 level were significantly lower than in younger patients. CD34+/CXCR4+ cells number: 2,8 (0,4–4,95) versus 5,7 (3,8–8,95); *P* < 0.0001 for patients ≥ and <50 years, respectively. SDF-1 level: 1,5 (0,6–2,4) versus 2,7 (1,4–3,4); *P* = 0.004 for patients ≥ and <50 years, respectively.

Plasma SDF-1 concentration was a single, independent prognostic factor of significant progenitor cells mobilization [Odds ratio (95% confidence interval): OR 5,8 (95% CI: 5–22); *P* = 0,01].

### 3.3. Left Ventricle Contractility and Remodeling

50 patients with acute MI were evaluated by echocardiography in order to determine the correlation between circulating progenitor cell number and LVEF and remodeling (ESD, EDD). Initial echocardiographic examination revealed LVEF impairment (≤40%) in 14 individuals (28% of MI patients). Significant LVEF improvement (≥5%) during the follow-up was observed in 19 patients (38%). In 1-year observation, decreased LVEF ≤40% was diagnosed in 19 patients (38%). There were no significant differences between median EDD and ESD values measured 12 months after MI and during the acute phase as shown in Table 3. Also no differences in terms of medical treatment between patients with and without recovery of LVEF were found.

### 3.4. Mobilization of the Stem and Progenitor Cells in Relation to LVEF and Remodeling

**Figure 4:**
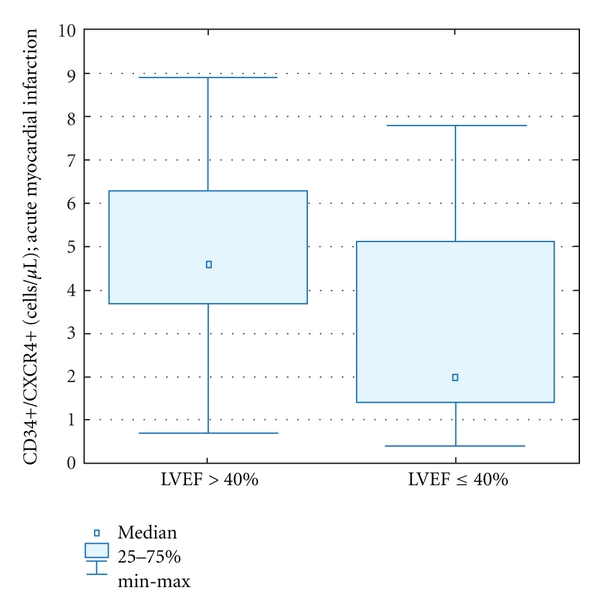
Circulating CD34+CXCR4+ cells in the acute phase of MI in patients with LVEF >40% versus ≤40%.

**Figure 5:**
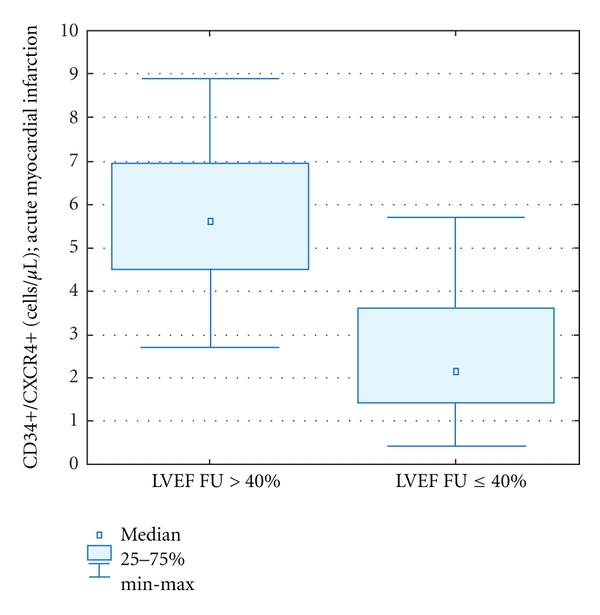
Mobilization of CD34+CXCR4+ cells in acute MI in patients who had preserved or reduced LVEF at 1-year followup post MI.

The absolute numbers of CD34+CXCR4+ cells in acute MI was significantly lower in patients with decreased left ventricle ejection fraction (LVEF ≤40%) in the acute phase of MI [2,0 (0,4–7,8) versus 4,7 (0,7–8,9); *P* = 0,028], as well as in 1-year followup [2,3 (0,3–5,8) versus 5,5 (2,8–8,9); *P* < 0.0001] (Figures 4 and 5).

**Figure 6:**
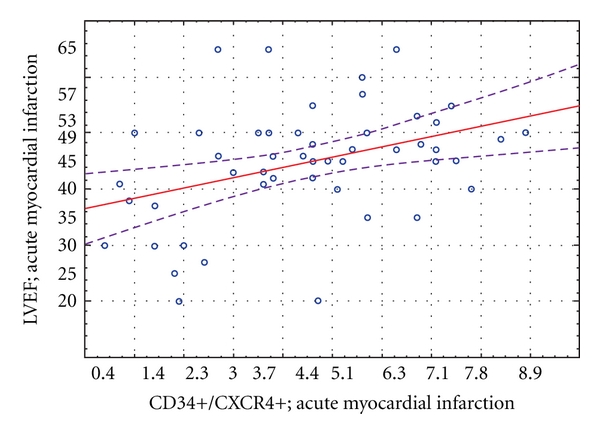
Correlation between CD34+CXCR4+ cells mobilization and initial LVEF value in patients with MI.

There was a significant positive correlation between CD34+CXCR4+ cells mobilization and left ventricle ejection fraction in first 24 hours after myocardial infarction (*r* = 0, 39, *P* = 0,03) (Figure 6).

In control echocardiographic evaluation it was shown that CD34+CXCR4+ cells number in acute MI was positively correlated with LVEF 12 months after MI (data not shown).

**Figure 7:**
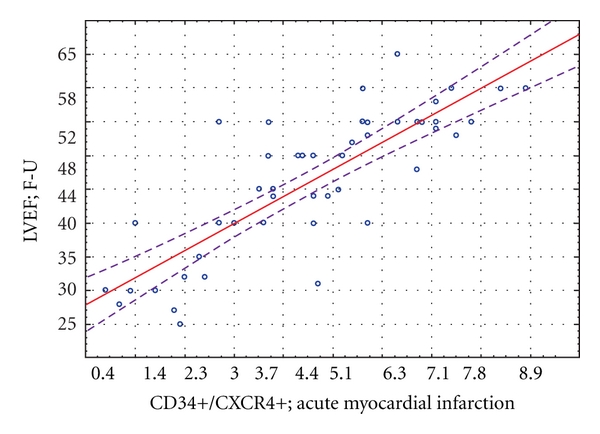
Correlation between peripheral blood CD34+/CXCR4+ progenitor cells number and the absolute LVEF change 12 months post MI.

There was significant positive correlation (*r* = 0, 41, *P* = 0,031) between the number of mobilized progenitor cells in the acute phase of myocardial infarction and LVEF change in 12-month observation (ΔLVEF; F-U) (Figure 7).

**Figure 8:**
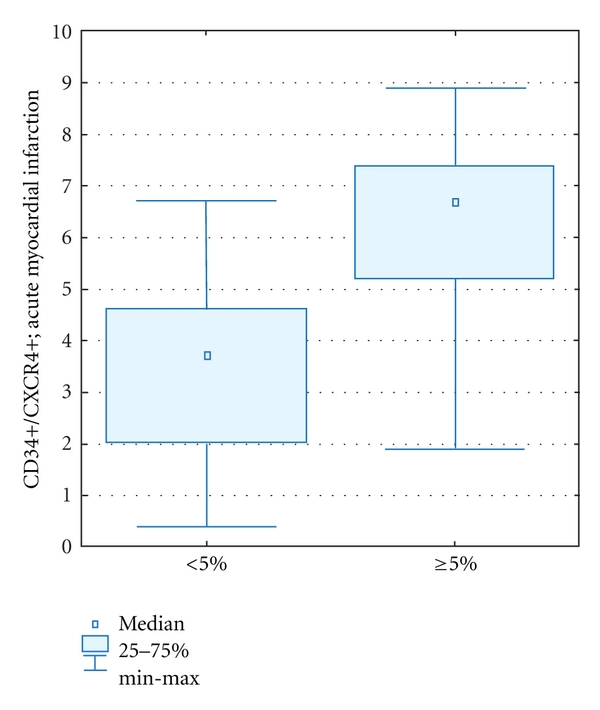
Comparison of CD34+/CXCR4+ progenitor cells mobilization in the acute phase of myocardial infarction in patients with the absolute LVEF improvement ≥5% (Δ ≥ 5%); 12-month clinical followup.

Additionally, it was shown that in patients who had LVEF increase ≥5% in 12-month observation the number of circulating cells in the acute phase of myocardial infarction was significantly higher comparing to patients with decreased LVEF, no LVEF change and insignificant LVEF improvement [6,8 (1,9–8,9) versus 3,7 (0,4–6,8); *P* < 0,0001] (Figure 8).

Multivariate logistic regression analysis included parameters which were predictors of changes of LVEF over 1 year in univariate model (peak values of TnI, peak activity of CK-MB, anterior localization of MI, time to reperfusion, and number of circulating CD34+CXCR4+ cells in acute MI). Only anterior localization of MI and peak values of TnI, but not number of circulating cells, were independent predictors of LVEF changes over time.

**Figure 9:**
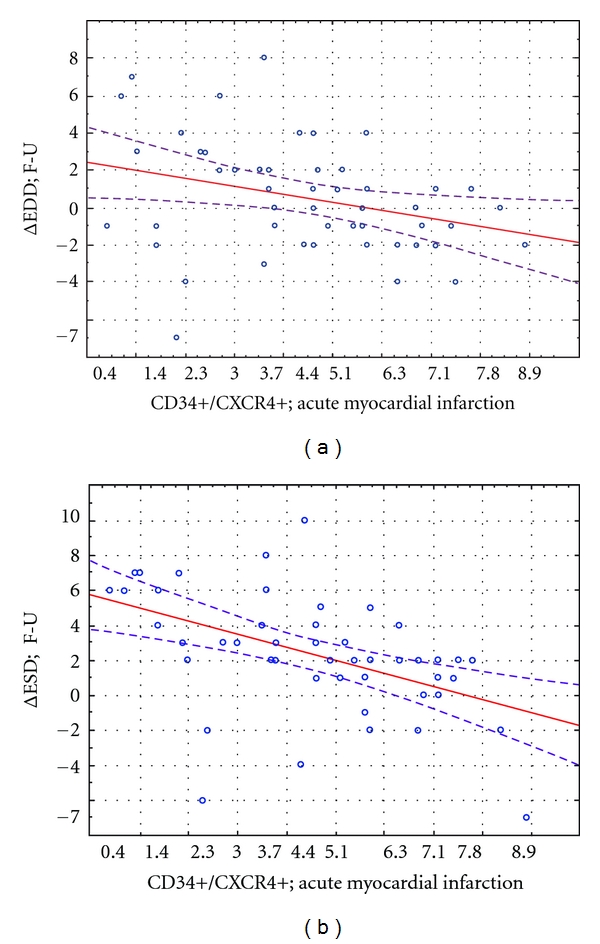
Correlation of CD34+CXCR4+ progenitor cell number in the acute phase of MI with the absolute EDD change (ΔEDD) and ESD (ΔESD) in 12-month clinical observation.

There was a significant negative correlation between mobilization of CD34+CXCR4+ cells in acute MI with ESD and EDD in the acute phase of MI as well as after 1 year (data not shown). The number of mobilized CD34+CXCR4+ cells in acute MI was inversely correlated with absolute change of ESD and EDD (Figure 9).

**Figure 10:**
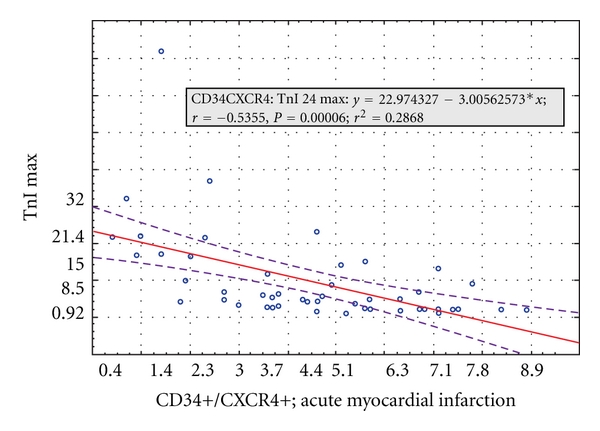
Correlation between CD34+CXCR4+ cells mobilization in the acute phase of MI and maximum levels of troponin I.

In MI patients with LVEF below 40% the SDF-1 levels were lower than in patients with preserved LVEF [1,45 (0,6–2,9) versus 2,0 (0,9–3,45) pg/mL; *P* = 0.008] (data not shown). There were no significant correlations between chemoattractants and LVEF and LV remodeling after 1 year. Mobilization of CD34+CXCR4+ cells measured in acute MI was negatively correlated with maximum TnI levels (Figure 10).

**Figure 11:**
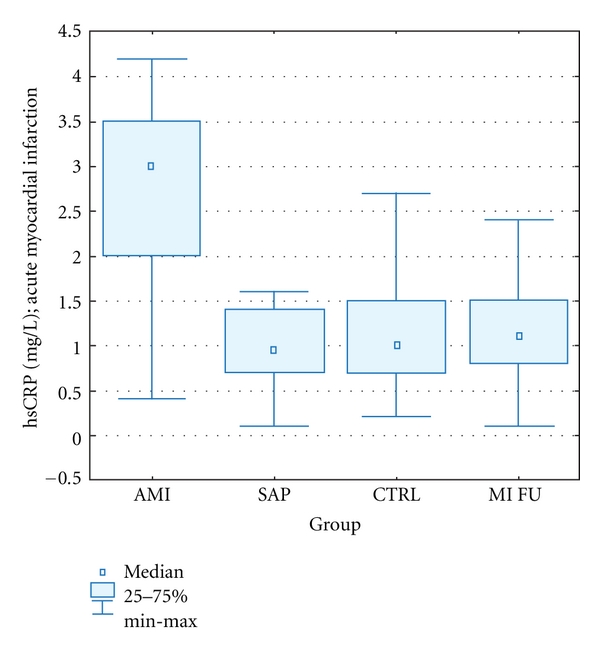
Plasma hsCRP levels. Patients: with acute myocardial infarction: AMI, with stable angina pectoris: SAP, control group: CTRL, 1-year followup: MI FU. *P* = 0,47 SAP versus CTRL; *P* < 0,0001 AMI versus CTRL; *P* < 0,0001 AMI versus SAP; *P* = 0,21 MI F-U versus CTRL; *P* = 0,73 MI FU versus SAP; *P* < 0,0001 MI F-U versus AMI.

NT-proBNP levels in patients with acute MI were significantly higher than in SA group [170 (34–860) versus 80 (23–167) pg/mL; *P* < 0,0001]. Number of circulating cells in acute MI was significantly negatively correlated with plasma NT-proBNP levels (*r* = −0,48, *P* = 0.03). In patients with MI, high sensitive C-reactive protein (hsCRP) level was statistically significantly increased. In 1 year follow-up, the hsCRP level were similar in all three groups (Figure 11).

There were no significant correlations between hsCRP levels and mobilization of CD34+CXCR4+ in acute MI (*r* = −0.17, *P* = 0.22).

### 3.5. Ergospirometry and Functional Status

The ergospirometry test was carried out in 48 of 50 patients after 1 year after MI. Results are shown in Table 4.

Only variable that correlated with mobilization of CD34+CXCR4+ cells was VO_2_ peak (**r** = 0.34, **P** = 0.01). Similarly, there were no differences in number of circulating cells and chemoattractants in patients stratified according to NYHA class at 1-year followup.

## 4. Discussion

Acute MI triggers the release into peripheral blood of BM-derived stem and progenitor cells, such as EPCs, VSELs, HSCs, and MSCs. In present study we provided evidence that mobilization of CD34+CXCR4+ cells in acute MI was significantly correlated with improvement of LVEF and LV remodeling in 1-year followup. Reduced mobilization of CD34+CXCR4+ cells in acute phase of MI was associated with more significant impairment of LVEF and greater infarct size measured as the release of TnI. We evaluated the mobilization of CD34+CXCR4+ cells in acute MI in comparison to patients with stable angina and control group without CAD. According to our previously published results the maximum mobilization of cells occurred early within 12 hours after the onset of ischemia and the number of circulating cells increased approximately 2.3-fold. The number of these circulating cells after 1 year was comparable to patients with stable CAD and healthy subjects. Mobilization of the stem and progenitor cells might therefore be considered a part of an inflammatory response in response to myocardial ischemia and necrosis. Current observations are consistent with other studies investigating mobilization of EPCs and HSCs which showed rapid release of cells. Therefore measurement of CD34+CXCR4+ cells at admission reflects in our opinion the maximum mobilization triggered by acute MI [3–5].

Our previous studies demonstrated that CD34+CXCR4+ and c-met+ cells are present in increased numbers for first 2-3 days and is gradually reduced within a week [4]. Accordingly Leone et al. showed that in acute MI several populations of cells CD34+CD33+, CD34+CD38+, CD34+CD117+, and CD34+VEGFR2+ are mobilized within 6 hours after the onset of ischemia and returned to levels comparable with stable CAD within 2 months [17]. Leone et al. also confirmed the rapid release of cells within 24 hours after MI [17]. Conversely, Shintani et al. showed that the peak number of CD34+ cells occurred later, about 7 days after the onset of ischemia [3]. Mobilization of BM cells was confirmed also in non-ST-segment elevation acute coronary syndromes [13].

So far there was no prospective study which investigated the relations between mobilization of BM cells and recovery of LV contractility following acute MI. In the present study we showed that the mobilization of CD34+CXCR4+ cells is positively correlated with LVEF both measured in the acute phase as well as its recovery over 1-year followup. We also showed that almost 30% of patients had reduced LVEF ≤ 40% and these patients had significantly less circulating cells than patients with preserved LVEF. Reduced mobilization was also observed in patients with no significant improvement of LVEF following reperfusion over the long-term followup. Patients with a significant increase of LVEF defined as increase ≥5% had significantly higher number of circulating cells. In addition our study suggests that the release of CD34+CXCR4+ cells is inversely correlated with LV remodeling measured by absolute increase of EDD and ESD over 1 year. Overall the findings show consistently that in patients with reduced LVEF and lack of significant improvement of contractility as well as more significant remodeling the mobilization of CD34+CXCR4+ cells in acute MI is reduced.

There is a paucity of data on such associations in the literature. Leone et al. assessed the correlation of CD34+ with LVEF in 54 patients with acute MI. Number of cells was measured after 1 year. Authors showed that mobilization of CD34+ cells was an independent predictor of improvement of LVEF and it was positively correlated with absolute increase of LVEF and negatively with wall motion score index (WMSI) and end-systolic volume. Patients with most significant improvement of LVEF as well as a reduction of WMSI and LV volumes had also higher number of circulating CD34+ cells 1 year after MI [16]. In this study however only 16 patients were treated with primary PCI and 12 had no reperfusion treatment at all. Also only 45% of cells were CXCR4+, so this study evaluated different population of BM cells [17]. Conversely Massa et al. showed no correlation between EPCs, HSCs, and LVEF in patients with acute MI [18]. In the present study we enrolled only patients treated with primary PCI and final TIMI3 flow to reduce the bias caused by inclusion of patients without proper reperfusion which translates into LV remodeling. We did not find any differences in mobilization of cells in subgroups of patients stratified by sex, presence of diabetes, hypertension, and obesity. Interestingly we observed that the mobilization was lower in older patients in comparison to those younger than 50 years. Proper interpretation of the results requires the consideration of other factors that can modulate the process of mobilization, including age, medications, and profile of CAD risk factors. Generally the mobilization and function of circulating SPC is reduced in elderly and diabetes. On the other hand statins improve the mobilization, viability, and function of these cells; however most available data referred to EPCs. Our population of cells was distinct from EPCs and definitely more heterogenous. On the other hand subpopulation of CXCR4+ cells (CD133+CXCR4+ VSELs) is indeed reduced in diabetic patients with acute MI [5, 13].

The presence of correlations between LV contractility, remodeling, and mobilization of cells which theoretically might contribute to tissue repair is clearly not a proof of a causal relationship but only a hypothesis-generating concept. We hypothesized that some populations of BM cells might be particularly intriguing because of their ability to express early cardiac and endothelial lineage markers which suggest they might play a role in cardiac reparatory reaction following MI. We previously showed that some populations of circulating cells (CD34+CXR4+, VSELS) express mRNA for early cardiac (Nkx2.5/Csx, GATA-4) muscle and endothelial markers (VE-cadherin, von Willebrand factor). The increased expression of these markers is in temporal correlation with maximum mobilization of CXCR4+ cells [4]. We also demonstrated that acute MI triggers mobilization of VSELs expressing early developmental markers (Oct-4, Nanog, SSEA-4). On the other hand, murine VSELS showed potential for differentiation into cardiac myocytes [6]. It seems that mobilization of CXCR4+ cells reflects not only an inflammatory reaction following MI, but is also a part of repair mechanism. Interestingly the potential for differentiation of circulating progenitor cells was shown to be correlated with improvement of LVEF after MI as shown by Numaguchi et al. [18]. In our study the levels of chemoattractants (SDF-1, G-CSF) were not significantly correlated with improvement of LVEF or remodeling. Release of TnI in acute MI is strongly correlated with the infarction size and is a predictor of LVEF recovery following MI. Other important predictors are anterior localization and time from the onset of symptoms to reperfusion [16]. In our hands the mobilization of CD34+CXCR4+ cells was inversely correlated to maximum levels of TnI and activity of CK-MB, which suggests that patients with blunted mobilization of stem cells developed larger infarcts. This might in part explain reduced LVEF recovery and increased remodeling in this group. Massa as well as Leone et al. however found no association between circulating cells and myocardial necrosis markers [5, 16, 17]. In addition Voo et al. showed that number of EPCs is positively correlated with myocardial necrosis and levels of CRP [19]. In multivariate analysis however the number of CD34+CXCR4+ cells was not an independent predictor of LVEF recovery.

We investigated if the mobilization of BM cells is related to functional status of the patients in 1-year follow-up. We showed no differences between patients presenting with MI with or without heart failure (Killip-Kimball class). In the long-term follow-up we used cardiopulmonary exercise test which is a noninvasive, reproducible, and reliable tool for evaluation of exercise tolerance. Such study was completed by 96% of patients. The only parameter which showed a positive correlation with the number of circulating cells measured 1 year after MI was VO_2_ peak. It seems that improved LVEF and less remodeling in patients with a higher number of circulating cells might have translated into better exercise capacity. NT-proBNP is a significant prognostic marker in acute coronary syndromes and heart failure [21]. We found increased levels of NT-proBNP in patients with MI which were inversely correlated with LVEF, but also with the number of circulating CD34+CXCR4+ cells. Valgimigli et al. showed that number of circulating CD34+CD45+ cells and EPCs in patients with chronic heart failure is reduced in higher NYHA class, inversely correlated with BNP and positively with peak VO_2_ in ergospirometry [21]. We found no evidence of correlation between cell mobilization, chemoattractants levels with NYHA and CCS class in 1-year followup. Several other studies showed that in patients with heart failure the numbers of circulating stem and progenitor cells as well as their functional capacity are reduced [15, 22].

Mobilization and homing of circulating stem and progenitor cells is regulated by expression of chemoattractants, such as chemokines (SDF-1), growth factors (VEGF) or cytokines (G-CSF) as well as activation of the complement cascade and bioactive phospholipids.

Several chemoattractants, such as SDF-1, LIF, and HGF, are expressed in the myocardium in particular in infarct border-zone. This suggests that cells with receptors for chemoattractant are preferentially taken up in these areas [22, 23]. Our study showed increased levels of G-CSF and decrease in SDF-1 levels in acute MI. Previously we demonstrated that following MI the baseline low levels of SDF-1 increase over time, which corresponds with peak expression of SDF-1 in the myocardium which occurred later than mobilization of CXCR4+ cells [7]. SDF-1 levels are predictors of significant mobilization of CD34+ cells [4]. Probably for stem cell homing the local expression of SDF-1 in the heart is more important than blood levels which show high individual variability. We found however no significant correlations between plasma G-CSF levels and cell mobilization as well as any of the clinical parameters. Similarly there were no significant correlations between increased levels of hsCRP in patients with acute MI and cell mobilization.

The limitations of the study are relatively small sample size and use of echocardiography instead of MRI in particular in the analysis of comparison between patients with and without significant (>5%) improvement of LVEF. Additionally, we compared the release of SPC in acute MI to number of cells in patients with stable CAD and control subjects without CAD. Latter two groups were different in regard to age and use of medications from patients with MI, so we could not have excluded the bias, because both factors modulate the number of circulating cells. 

### 4.1. Summary

Mobilization of CD34+CXCR4+ cells in acute MI shows significant positive correlation with left ventricular ejection fraction and inverse correlation with infarct size and NT-proBNP levels. Number of circulating cells is lower in patients with reduced LVEF, LV remodeling and those without significant improvement of LV contractility in 1-year follow-up.

## Figures and Tables

**Table 1:** Characteristics of the study group.

	CTRL (*n* = 20)	SAP (*n* = 28)	AMI (*n* = 50)	*P*
Age (years) (mean ± SD)	44,6 ± 6,2	56,7 ± 11,6	58 ± 11,5	*P* < 0.05 versus CTRL
Age (years) (median ± IQR)	44 (34–54)	56 (32–75)	57 (30–79)	*P* < 0.05 versus CTRL
Male, *n* (%)	17 (57)	18 (60)	30 (60)	*P* = NS
Hypertension, *n* (%)	7 (23)	17 (57)	30 (60)	*P* < 0.05 versus CTRL
Hypercholesterolaemia, *n* (%)	8 (26)	23 (77)	35 (70)	*P* < 0.05 versus CTRL
Type 2 diabetes mellitus, *n* (%)	0	11 (36)	18 (36)	*P* < 0.05 versus CTRL
Smoking, *n* (%)	12 (40)	19 (63)	32 (64)	*P* < 0.05 versus CTRL
Family history of IHD, *n* (%)	8 (27)	13 (43)	24 (48)	*P* < 0.05 versus CTRL
Statins prior to hospitalization, *n* (%)	0	20 (67)	32 (64)	*P* < 0.05 versus CTRL
ACE inhibitors, *n* (%)	4 (13)	16 (53)	23 (46)	*P* < 0.05 versus CTRL
Acetylsalicylic acid, *n* (%)	2 (7)	28 (100)	31 (62)	*P* < 0.05 versus CTRL, SAP
Total cholesterol [mg/dL]	199 (156–256)	201 (156–256)	201,5 (122–313)	*P* = NS
HDL cholesterol [mg/dL]	43 (20–70)	43 (24–75)	41 (13–74)	*P* = NS
LDL Cholesterol [mg/dL]	97 (89–124)	100 (65–216)	105 (65–113)	*P* = NS
Triglycerides [mg/dL]	150 (123–200)	176,5 (84–269)	163 (76–375)	*P* = NS
Creatinine [mg/dL]	0,9 (0,7–1,4)	0,9 (0,8–1,3)	0,9 (0,7–14)	*P* = NS
Erythrocytes [×10^6^/*μ*L]	4,7 (4,2–5,1)	4,7 (4,3–5,1)	4,62 (4,12–5,2)	*P* = NS
Leucocytes [×10^3^/*μ*L]	6,9 (5,5–8,3)	6,6 (5,2–7,4)	10,17 ± 2,8	*P* < 0.001 versus CTRL, SAP
Monocytes [×10^3^/*μ*L]	0,8 (0,4–1,2)	0,7 (0,46–1,14)	0,76 (0,41–1,1)	*P* = NS
Platelets [×10^3^/*μ*L]	195 (143–246)	194 (137–251)	198 (146–250)	*P* = NS

Initial LVEF ≤40%, *n* (%)	—	—	14 (28)	
Initial CKMB [U/l]	—	—	26,5 (5–136)	
Initial TnI [ng/mL]	—	—	0,7 (0,0–18)	
Maximal CKMB [U/l]	—	—	109,5 (5–572)	
Maximal TnI [ng/mL]	—	—	4,7 (0,92–72)	
Anterior wall infarction, *n* (%)	—	—	30 (60)	
Multivessel CAD, *n* (%)	—	—	26 (52)	

**Table 2:** 

	CTRL	SAP	AMI	MI F-U (1 year)
SDF-1 [pg/mL]	3,2 (0,2–4,4)	2,9 (0,1–4,4)	1,8 (0,6–3,4)	3,0 (0,1–4,2)
*P*		0,77 versus CTRL	<0.0001 versus CTRL <0,0001 versus SA	0,81 versus CTRL 0,92 versus SA <0,0001 versus MI
G-CSF [pg/mL]	27 (0,7–66)	25 (0,1–50)	74 (6–141)	30 (0,8–71)
*P*		0,78 versus CTRL	<0,0001 versus CTRL <0,0001 versus SA	0,9 versus CTRL 0,83 versus SA <0,0001 versus MI

**Table 3:** Left ventricle ejection fraction and remodeling in 1-year follow-up in patients with MI.

Parameter	Acute MI	1 year FU	*P* value
LVEF (%)	44,7 ±10,2	45,9 ± 10,5	0,17
EDD (mm)	51,6 ± 5,5	51,1 ± 5,5	0,9
ESD (mm)	33,4,6 ± 3,9	35,1 ± 5,1	0,08
ΔLVEF (%)	—	1,2 ± 6,7	—
ΔESD (mm)	—	2,5 ± 3,3	—
ΔEDD (mm)	—	0,5 ± 2,9	—

**Table 4:** Results of ergospirometry.

	VO_2_ peak	VE/VCO_2_ slope	VE/VCO_2_ peak/rest	VE/VCO_2_ peak	VE/VCO_2_ rest
Median (Range)	26.0 (13–33)	29.7 (18,1–41,6)	0.88 (0,68–0,99)	33.13 (24–50)	42.98 (30–51)
